# Biased media? How news content influences age discrimination claims

**DOI:** 10.1007/s10433-018-0465-4

**Published:** 2018-03-10

**Authors:** Anne Cornelia Kroon, Damian Trilling, Martine Van Selm, Rens Vliegenthart

**Affiliations:** Universiteit van Amsterdam, Amsterdam, Netherlands

**Keywords:** Media stereotypes, Older workers, Time series, Age discrimination claims

## Abstract

Information distributed via the news media is acknowledged as a potential source of negative beliefs about, and biased behaviors toward, older workers. Focusing on the Netherlands, the current study explains age discrimination claims filed by older workers by investigating the impact of visibility and media stereotypes of older workers in the news media, while controlling for real-world events and older workers’ expectations of unemployment (2004–2014). The results, based on time-series analysis, reveal that the visibility of older workers in the news media is associated with higher levels of age discrimination claims. This effect can be partly explained with the visibility of the negative media stereotype that older workers experience health problems in the content of news coverage. Furthermore, unemployment expectations decreased the number of age discrimination claims. These results offer support for the notion that the news environment is a source of variation in the experience of age discrimination at the workplace.

## Introduction

Equality in employment is one of the core labor market principles of the European Union. Yet, the experience of prejudice and discrimination is a common reality in the lives of senior members of the workforce in the EU (Abrams et al. 2011; Andriessen et al. 2014). Cumulating evidence suggests that older workers experience unequal access to employment, training, promotion, as well as job retention, with negative consequences for individual career prospects, life quality, and health (Abrams and Swift 2007; Bal et al. 2011; Finkelstein et al. 2013). The implications of these findings are alarming, particularly in light of the current aging of workforces, and signal the importance of understanding the factors that trigger age discrimination.

The limited body of the literature that addresses variation in the experience of age discrimination at the workplace has offered mostly static explanations based on experimental or cross-sectional data. The experience of discrimination is, however, not a stable process, but instead varies across time and as a consequence of contextual factors (Rippon et al. 2015). Previous studies investigating variation in prejudice and discrimination over time have focused on the context of minority groups and public attitudes and show that public opinion and real-world developments affect anti-minority sentiment and support for discrimination (Boomgaarden and Vliegenthart 2009; Coenders and Scheepers 1998). Older workers cannot be considered a minority group, yet; the categorization between “older” versus “other” or “younger” workers elicits group-based bias, which may be affected by contextual cues in a comparable manner.

In addition to mapping the influence of exogenous events and public opinion data, the current study includes media coverage as an exogenous variable explaining age discrimination claims. A long-standing history of research has consistently demonstrated that media portrayals of diverse groups in society can be biased and have the potential to promote the reliance on stereotypes when making decisions regarding, for example, whom to hire or fire (e.g., Ramasubramanian 2011; Ramasubramanian and Oliver, 2007). In the specific case of older workers, previous research finds that news media portray this group in terms of dominant stereotypes (Kroon et al. 2016b) and that exposure to such media stereotypes leads to a bias in perceptions and selection of older workers (Kroon et al. 2016a).

Particularly, news media are powerful distributors of stereotypical images, as information in news stories can be diffused via online and offline interpersonal communication or picked up by other media (Boomgaarden and Vliegenthart 2009; Bright 2016), and reach individuals that were not initially exposed to the content. Moreover, news media have been shown to “set” the agenda of other media types, meaning that the content of, for example, television is heavily influenced by news media coverage (e.g., Schuck et al. 2013).

The current study addresses the question whether variation in news coverage about older workers affects the filing of age discrimination claims by older workers in the Netherlands. The study considers news media coverage in terms of (a) the *visibility* of older workers (i.e., news media’s references to older workers) and (b) dominant *media stereotypes* (i.e., news media’s stereotypical references to the skills and qualities of older workers). The study’s dependent variable of interest is the number of formal complaints filed by older workers in the Netherlands regarding the experience of employment-related discrimination. The study covers a 10-year period (2004–2014) using quarterly data.

The current study contributes to the understanding of the relationship between media coverage and age discrimination claims over time. Previous research offers mainly cross-sectional explanations for variations in biased behavior based on research conducted in a laboratory setting, such as under which conditions employers select younger over older candidates for hiring or participation in job training programs (Krings et al. 2011; North and Fiske 2016). However, researchers have warned that the over-reliance on experimental methods in the domain of bias and prejudice “creates a theoretical echo chamber in which ideas are not cross-fertilized by research conducted in real-world settings” (Paluck and Green 2009). To address this concern, the present study relies on an unobtrusive measurement of discrimination (i.e., discrimination litigation) and traces variation on the aggregate level over time.

## Age discrimination and news coverage

The current study departs from the assumption that discrimination claims are an indicator of actual discriminatory events faced by older workers (see Wakefield and Christopher 2004). Filing a discrimination claim is a long and rather stressful process (Wakefield and Christopher 2004), and therefore, it is unlikely that individuals are willing to initiate this taxing process without the actual experience of being discriminated. Empirical evidence demonstrates that perceptions of fairness and justice indeed predict the filing of legal discrimination claims (Goldman et al. 2006; Goldman 2001, 2003).

Scholars have identified media representations of older adults as a source of deeply rooted negative societal beliefs about, and biased behavior toward, older workers (Abrams et al. 2015; Kessler et al. 2004, 2010; Kotter-Grühn 2014). The current study considers the influence of both the *visibility* of older workers in the news, and *media stereotypes* about older workers. Common negative stereotypes that are present in Dutch news media relate to older workers’ poor health and low productivity, while positive media stereotypes relate to older workers knowledge, experience, and reliability (Kroon et al. 2016b). In the current study, discrimination claims are traced among older workers aged 45 years and older, as from this age limit on it becomes increasingly difficult for individuals to reenter the labor market after job loss in the Netherlands (Bierings and Loog 2013).

The question is then how the visibility of older workers in the news media may affect the filing of age discrimination claims. We expect a positive relationship between older workers’ media visibility and age discrimination claims for three reasons. First, it is assumed that news about older workers is generally negative in nature. Previous research indicates that older workers feature in news stories addressing the problems faced by this group on the labor market, such as long-term unemployment and the experience of age discrimination (see Kroon et al. 2015).

Second, we assume that news coverage about older workers creates opportunities for negative social comparison, as “news media can influence people’s readiness to categorize others” (Boomgaarden and Vliegenthart 2009). The literature on age-group categorization suggests that people use age-group categories, such as “young workers,” “middle-aged workers,” and “older workers” to categorize themselves and others (Bytheway 2005). Following Social Identity Theory, these categorization processes affect how we think about others and ourselves, between “us” and “them” (Taijfel and Turner 1979). When older workers are salient in the media environment, this may remind people of their distinct identities and highlight perceived differences with older workers.

Third, it is assumed that the effect of negative news coverage about older workers outweighs the effect of positive news coverage about older workers. We base this assumption on evidence for the “negativity bias.” In short, the notion of “negativity bias” describes the tendency of individuals to weigh negative information more heavily than positive information (Soroka 2006). This implies that negative news content is likely to have a greater and longer lasting impact than positive news content (Soroka and McAdams 2015). Empirical work confirms that public responses to negative information are much greater compared to public responses to positive information (e.g., Soroka 2006; Soroka and McAdams 2015).

In sum, we can assume that news about older workers is negative and offers opportunities for social categorization and comparisons and that negative effects likely outweigh positive effects. Based on this literature, we expect that increased visibility of older workers in the news media will exert a negative influence on perceptions of older workers, which in turn will create opportunities for age discrimination. Under these circumstances, the volume of age discrimination claims will increase. We hypothesize:

### **H1**

The visibility of older workers in the news will positively affect the number of age discrimination claims filed by older workers.

In addition to the visibility of older workers in the news media, our study investigates the influence of media stereotypes on the filing of age discrimination claims. Ample evidence suggests that stereotypical inferences have a persuasive effect on employers’ and employees’ ability to make fair judgments regarding older workers (Krings et al. 2011). In addition, age stereotypes can be considered tools that help justify biased behavior (Finkelstein et al. 2000).

Stereotypes about older workers are mixed in terms of valence (Bal et al. 2011; Bowen and Skirbekk 2013; Shiu et al. 2015). Positive dispositions relate to older workers’ “soft” skills, in particular their assumed loyalty and reliability (Bal et al. 2011), while negative dispositions relate to “hard” skills, such as low physical capacity to deal with workload (i.e., problematic health status), competence, and productivity (Bal et al. 2011; Van Dalen et al. 2010; Posthuma and Campion 2009). As was mentioned before, previous research indicates that these positive and negative stereotypes are reproduced by the news media (Kroon et al. 2016b).

Media stereotypes might influence the filing of age discrimination claims. Media stereotypes have the power to shift beliefs in the direction of the portrayals and to generate stronger biased beliefs (e.g., Arendt 2013; Kroon et al. 2016a; Ramasubramanian 2011; Seate and Mastro 2017). This, however, does not mean that positive media stereotypes are equally powerful as negative media stereotypes. The effects of negative stereotypes on individuals’ perceptions of older workers are (much) stronger; when exposed to mixed-media stereotypes, the effect of the negative stereotype component outweighs the positive component, resulting in a negative net effect (Krings et al. 2011; Kroon et al. 2016a). In sum, experimental data demonstrate that negative media stereotypes, relative to positive media stereotypes, are especially powerful in fostering discrimination in the workplace via bolstered negative stereotypical believes about older workers’ skills and capacities (Kroon et al. 2016a).

No direct empirical evidence exists for the relationship between exposure to media stereotypes and the filing of age discrimination claims over time. Based on the available evidence, it is anticipated that negative (vs. positive) media stereotypes about older workers will exert a stronger effect on the incidence of discrimination in the workplace (Kroon et al. 2016a). The level of discrimination, in turn, is likely connected to the volume of discrimination claims (Wakefield and Christopher 2004). Therefore, we expect:

### **H2**

The positive effect of the number of negative media stereotypes on the volume of age discrimination claims filed by older workers will be stronger than the negative effect of positive media stereotypes.

## Method

The study relies on the period from the second quarter (q2) of 2004 till the second quarter (q2) of 2014, as for this time frame discrimination claims were available. The data were provided by The Netherlands Institute for Human Rights (NIHR). When Dutch citizens experience discrimination, they can start a procedure by filing a discrimination claim to NIHR, after which an investigation and possible legal proceedings will be set in motion. In the research period, the NIHR dealt with 437 discrimination claims on the basis of age in the domain of employment made by people between 45 and 64 years of age, compared to 166 discrimination claims made by people younger than 45 years of age. Of 289 people, age was not registered.

The dependent variable “age discrimination claims” was computed by taking the quarterly number of claims made by older workers (45–64 years of age). We rely on the moment that an individual older worker files a complaint to the NIHR via email or telephone. This is most closely related in time to the actual experience of age discrimination in the workplace and therefore preferable to the date of the legal judgment (which causes a delay of up to 6 months). Thirty-three claims were removed because the moment that the claim was filed was not available. The final number of discrimination claims is 404, with an average of 9.61 age discrimination claims per quarter (*SD* = 0.62).

To explain variation in these discrimination figures, we make use of the following data types: exogenous events, public opinion data, and media content data. To start, two exogenous key events were identified: *the financial crisis* and the *debate about the state pension age*. We control for these events, as we are interested in the effect of media coverage of older workers on the filing of age discrimination claims, above and beyond that of specific time frames. First, the financial crisis marks a period in which workers of all ages may have felt more threatened in their job, with possible consequences for the likelihood that they feel and report being discriminated. A dummy variable was included capturing the time frame of the financial crisis (2008q1–2010q4). Second, the history of the debate leading toward the formal postponement of the retirement age can be characterized as being fairly turbulent. A second dummy was added capturing the key events in the debate about the postponement of the retirement age (coded as “1”):

- The period 2008q4–2009q4, capturing the initial phase of the debate about the postponement of the state pension age. In this period, two draft laws aimed at a more flexible and higher retirement age were proposed.
- The period 2011q2–2011q3. During this timeframe, the previously proposed law was withdrawn after being declared as controversial, and a new proposal was introduced.
- The period 2012q2–2012q3, capturing the moment that the final law proposal was introduced and approved by the Dutch parliament.

Next, we move to our public opinion data. Previous research has identified unemployment rates as an important determinant of discrimination claims (Donohue and Siegelman 1991; Wakefield and Christopher 2004). Our measure of expected unemployment is obtained from Statistics Netherlands. Each month, a thousand Dutch households are asked to answer a set of questions regarding the economy in general as well as people’s individual financial situation (Statistics Netherlands 2017). Expected unemployment was measured with the following question: “How do you think the unemployment in the Netherlands will develop in the next 12 months? Will it, according to you, go up, go down, or remain the same?” (*1* = *clearly fall, 5* = *clearly rise*). For the current study, only the answers of participating household members in the age category 45–65 were selected. The mean level of respondents’ answers was computed and varies on the quarterly level (*M* = 3.63, *SD* = 0.10).

Last, we describe our media variables. For the research period, all news articles referring to older workers published in the five largest Dutch national newspapers were retrieved: de Volkskrant, De Telegraaf, Trouw, Algemeen Dagblad, and NRC Handelsblad. The following search string was used: “older worker* OR older employee*”. This resulted in a final sample of 2123 news articles.

Second, a weighted score for older workers’ visibility was created: News articles that refer more frequently to older workers are assigned a higher score and news articles that mention older workers at the beginning of the news article weight more heavily than articles that refer to older workers at the end of the article. Specifically, the following equation is used to compute our measure of older workers’ visibility:

$$v({\text{visibility}}) = \mathop \sum \limits_{a \in articles} ((\ln (n\;{\text{referrals}}\;{\text{search}}\;{\text{terms}})) \times (\ln ({\text{position}}\;{\text{first}}\;{\text{search}}\;{\text{term}})) / n\;{\text{words}}) \times 100 ,$$

where *v*(visibility) is the visibility of older workers in a news article. The score is dependent upon the number of referrals to the search terms (i.e., “older worker*” OR “older employee*”) in both the headline and the body of the text (ln(referrals search terms)). The number of referrals to older workers adds sublinearly to their visibility within a specific news article. Second, the score is made dependent upon the proportional position of the first referral (ln(position first keyword)), so that the first word of the article is assigned a weight of (ln(100)), and the last word as (ln(1)) (see Boomgaarden et al. 2010; Boomgaarden and Vliegenthart 2007).^1^ The relative scores were aggregated to the quarterly level (*M* = 72.22, *SD* = 4.73).

Next, we move to our media stereotype measures. The measurement instrument was developed in two steps. First, four distinct and dominant media stereotypes about older workers were identified on the basis of previous research (Bal et al. 2011; Kroon et al. 2016a, b): “problematic health status” and “low productivity” (negative); as well as “reliable and involved” and “experienced” (positive). These are the most common media stereotypes of older workers in Dutch news media (Kroon et al. 2016a, b).

In a second step, for all four media stereotypes, keywords were identified to measure the presence of the respective stereotypes in the news. “problematic health status” is indicated by unhealthy, physically weak, tiredness, lack of energy. “unproductive” is indicated by unproductive, slow, sluggish, inattentive, apathetic, passive, depreciated, incapable, and unmotivated. “reliable and involved” is indicated by reliable, involved, honest, loyal, and collegial. “experienced” is indicated by experience, knowledge, and wisdom.

The media stereotypes were measured using computer-assisted content analysis. We wrote a Python script to identity whether references to older workers occurred in the same sentence with one of the above-listed keywords in the entire sample of news articles. This was done with the use of regular expressions. Regular expressions can be seen as a sequence of characters that help to define a search pattern in texts, and are particular effective in setting the conditions for rather complex search combinations. First, regular expressions were used to capture different conjugations of a word.^2^ Second, regular expressions were used to set the allowed distance between words.^3^ Last, regular expressions were used to set the conditions for words that were not allowed to occur in a specific sentence in order to capture a stereotype.^4^ Each time a match was found, the relative position of the match within the specific news article was saved and written to the output.

In a final step, the score of negative and positive stereotypes is weighted upon their frequency and position within news articles. For each article, the number and position of referrals to the search terms were obtained to capture the visibility of stereotypes within articles. The same equation used to compute the visibility of older workers was used to calculate the visibility of the four stereotypes:

$$v({\text{stereotype}}) = \mathop \sum \limits_{{a \in {\text{articles}}}} ((\ln (n\;{\text{stereotype}}\;{\text{referrals}})) \times (\ln ({\text{position}}\;{\text{first}}\;{\text{keyword}})) / n\;{\text{words}}) \times 100$$

where *v*(stereotype) is the visibility of a specific stereotype within in a certain text. The score is dependent upon the number of referrals in both the headline and the body of the text (ln(stereotype elements)) and the proportional position of the first stereotypical referral ln(position first keyword) (ln(position first keyword)). Again, when keywords referring to media stereotypes are used more frequently, one additional single term adds less. The scores were aggregated to the quarterly level. Table 1 provides an overview of the design elements of the study, and Table 2 displays the bivariate correlations.

**Table 1:** Illustration for the design elements of the study

Type of data	Variables	Type of variable	Unit of analysis	Sampling	Type of data analysis
Exogenous events	Financial crisis	IV	Time frames	Census	Primary
	Debate about the pension age	IV	Time frames	Census	Primary
Public opinion data	Expected unemployment	IV	Employed and unemployed members from Dutch households in the age category 45–65 years		Secondary (Primary source: Statistics Netherlands)
Media content data	Older workers’ visibility	IV	Media coverage	Census (analysis of all coverage in the national newspapers)	Primary
Media content data	Older workers’ media stereotypes	IV	Media coverage	Census (analysis of all coverage in the national newspapers)	Primary
Age discrimination claims	Age discrimination claims	DV	All older workers and job seekers in the Netherlands in the age category 45–64 years of age	Census (all Dutch older workers/job seekers are eligible to submit a discrimination claim)	Secondary [primary source: The Netherlands Institute for Human Rights (NIHR)]

*IV* independent variable, *DV* dependent variable

**Table 2:** Bivariate correlations

	1	2	3	4	5	6	7	8	9	*M*	*SD*
1. Age discrimination claims	1									9.619	0.661
2. Crisis	0.094	1								0.286	0.071
3. Postponement retirement age	0.088	0.312*	1							0.214	0.064
4. Expected unemployment	− 0.292^†^	0.163	0.408**	1						3.629	0.098
5. Visibility older workers	− 0.224	− 0.067	− 0.022	0.153	1					72.221	4.731
6. *Neg*_*MS*_ : problematic health status	− 0.113	− 0.066	− 0.160	0.155	0.501**	1				1.266	0.246
7. *Neg*_*MS*_: unproductive	− 0.065	0.153	0.110	0.190	0.338*	0.125	1			1.695	0.273
8. *Pos*_*MS*_: reliable	− 0.087	− 0.028	0.055	0.205	− 0.035	0.327*	− 0.137	1		0.481	0.118
9. *Pos*_*MS*_: experienced	− 0.199	0.094	0.087	0.316*	0.656***	0.636***	0.251	0.474	1	3.039	0.451

*Neg*_*MS*_ negative media stereotype, *Pos*_*MS*_ positive media stereotype

^†^*p* < 0.1,*p < 0.05, **p < 0.01, ***p < 0.001

### Analysis

For analysis, autoregressive distributed lag (ADL) techniques were used. STATA 13 was used for the data analysis. As the data are aggregated to the quarterly level, the specific characteristics of time-dependent data have to be taken into consideration. ADL models account for overtime variations by allowing the inclusion of lagged values of the dependent variable as well as current and lagged values of the explanatory variables. ADL is a suitable technique to model the data under investigation, as we deal with aggregated data and a relatively high number of time points (10.5 years × 4 quarters = 42 time points) (Boef and Luke 2008).

Several steps were taken to account for the specific time-series structure of the data. First, the series should be non-stationary; the mean should not be dependent on the time of observation. Augmented Dickey–Fuller test yields significant results for our dependent series, confirming stationary processes.^5^ Second, an autoregressive term (AR(1) component)) was added, representing the influence of the dependent series’ past values on the current value (*t* − 1). This means that we model the influence of discrimination claims of the previous quarter on the current values herewith accounting for the overtime dependency of the series, based on the idea that the current value depends on the previous value. After inclusion of the AR term, we attain a model with residuals that are white noise. The Ljung–Box Q test indicates that both residuals and squared residuals are nonsignificant for the specified models, indicating no autocorrelation in the residuals (see Table 3).

**Table 3:** Explaining age discrimination claims with exogenous events, public opinion data, and media variables

	Lags	Model 1: univariate model		Model 2: contextual model		Model 3a: media model		Model 3b: media model		Model 3c: media model		Model 3d: full model	
		B	SE	B	SE	B	SE	B	SE	B	SE	B	SE
AR(1)	1	0.307	0.149*	0.153	0.168	0.119	0.157	0.055	0.153	0.109	0.163	0.086	0.154
Crisis	0			0.352	1.467	0.202	1.350	0.723	1.323	0.640	1.517	1.286	1.415
Postponement state pension age	0			0.643	1.706	1.589	1.612	1.029	1.575	1.292	1.704	0.439	1.622
Expected unemployment	2			− 2.322	1.096*	− 2.857	1.032**	− 3.009	0.978**	− 2.820	1.112*	− 2.499	1.017*
Visibility of older workers	2					0.053	0.020*	0.032	0.026	0.068	0.032*	0.051	0.031
Neg_*MS*_: problematic health status	2							0.960	0.453*			1.431	0.539*
Neg_*MS*_: unproductive	2							− 0.345	0.383			− 0.260	0.382
Pos_*MS*_: reliable and involved	2									0.790	1.168	− 0.260	0.382
Pos_*MS*_: experienced	2									− 0.234	0.395	− 0.595	0.390
Constant		6.803	1.569***	16.531	4.816**	14.717	4.498**	16.880	4.348***	13.863	5.024*	14.312	4.574**
*N*		41		40		40		40		40		40	
*R* ^2^		0.098		0.1912		0.330		0.440		0.340		0.488	
AIC		233.587		229.805		224.260		221.121		227.651		221.486	
LBQ(*R*)		15.875		11.782		14.109		22.656		13.297		17.599	
LBQ(*R*^2^)		25.473		19.074		30.568		12.789		23.670		16.631	

*Neg*_*MS*_ negative media stereotype, *Pos*_*MS*_ positive media stereotype

*p < 0.05, **p < 0.01, ***p < 0.01

Several models were tested, adding the independent variables to the univariate ADL model step by step. Model fit was inspected using the Akaike information criterion (AIC), which corrects for the inclusion of independent variables. The AIC is useful in comparing the fit of the specified models. Here, lower indices indicate better fit. The explanatory power of the models was assessed using *R*^2^. As displayed in Table 2, the positive and negative media stereotype series are significantly correlated and therefore partly overlap. To avoid issues with collinearity, we include the series of the negative and positive stereotypes in separate models.

Before adding the independent variables to the model, the delay of the effects (lags) needs to be determined. The appropriate lag lengths are established statistically and a priori based on an analysis of the cross-correlation functions (CCF) of the independent and dependent variable. The analysis suggests an appropriate lag length of two quarters for expected unemployment, older workers’ visibility and stereotype visibility (see Table 3). By including the independent variables at a lag length of two quarters, the basic criteria of temporal consistency for causality are met: A change in the independent variable precedes changes in the dependent variables (Vliegenthart 2014). Consequently, the analyses bring us a step closer to assessing causality (for other studies analyzing media data using ADL modeling techniques, see Jonkman et al. 2016; Van der Meer 2016). In order to test the hypotheses, the significance level of the unstandardized coefficients in the ADL model will be inspected.

## Results

We now proceed to the statistical testing of the hypotheses. In the univariate model (Table 3, Model 1), only the AR(1) term was added. The term is significant, which indicates that the current volume of age discrimination claims depends on the volume of age discrimination claims in the previous quarter. The two exogenous events (financial crisis and postponement of the retirement age), as well as the expected unemployment, were added in the contextual model (Table 3, Model 2). The AIC decreases, indicating better model fit compared to the univariate model. The two exogenous events do not influence the number of age discrimination claims. This means that during these events the number of age discrimination claims filed by older workers did not alter significantly during these time periods. We do, however, find a significant relationship between the mean expected unemployment and the dependent variable’s series. The results show that the lagged values (*t* − 2) of expected unemployment negatively influence discrimination claims (*B* = − 2.32, *SE* = 1.09, *p* < 0.05). This finding indicates that one-unit increase in the mean expected unemployment leads to 2.32 less age discrimination claims six months later.

**Fig. 1:**
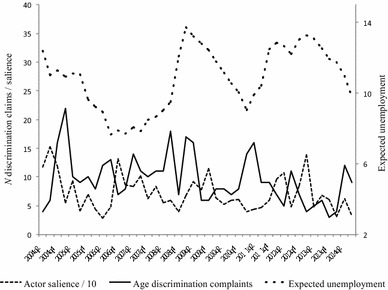
News media attention for older workers

Next, we turn to the first media model (Table 3, Model 3a). Here, we added the variable older workers’ visibility. We anticipated that increased visibility of older workers would increase the number of discrimination claims filed by older workers. The results offer support for this hypothesis: The lagged values (*t* − 2) of the series older workers’ visibility increase the number of discrimination claims (*B* = 0.05, *SE* = 0.02, *p* < 0.05). A one-unit increase in visibility leads to 0.05 more discrimination claims 6 months later, keeping other factors constant. This can be considered substantial given the variability of the variable’s series (see Fig. 1); a one *SD* change in visibility results in a 0.25 change in age discrimination claims. We find support for H1.

Next, the media stereotype variables were added to the model to test the hypothesis that the positive influence of media stereotypes on the filing of age discrimination claims by older workers is stronger for negative (vs. positive) media stereotypes. First, the series of the two negative media stereotypes were added (Table 3, Model 3b). AIC suggests that this is the best model under investigation. The model explains 44% of the variance in age discrimination claims. The results show that the effect of visibility becomes nonsignificant, while the negative effect of expected unemployment remains significant. The lagged values (*t* − 2) of the series visibility of the negative stereotype that older workers’ health is poor increase the number of discrimination claims (*B* = 0.96, *SE* = 0.45, *p* < 0.05). A one-unit increase in the visibility of this stereotype leads to 0.96 more age discrimination claims 6 months later. The series of the negative stereotype that older workers are unproductive did not exert an influence. Hence, we only find support for the negative effect of the negative media stereotype that older workers’ health is poor.

**Fig. 2:**
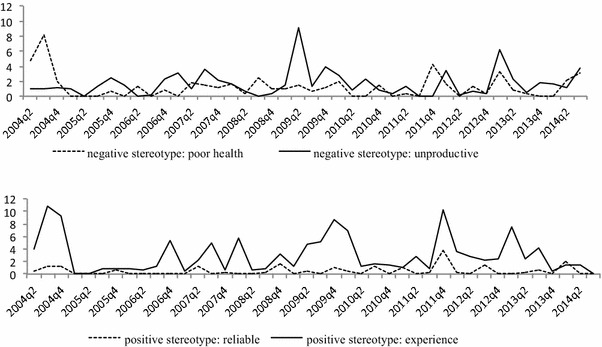
Negative (upper) and positive (lower) stereotypes about older workers

In Table 3, Model 3c, the negative media stereotypes are exchanged for positive media stereotypes. Both positive stereotypes do not exert an effect on the number of discrimination claims. The full model is displayed in Table 3, Model 3d, and confirms that the positive effect of the negative media stereotype that older workers face health problems remains significant after controlling for the positive media stereotypes. These results offer partial support for H2. Figures 1 and 2 display the series of the dependent and independent variables.

## Discussion

The current study investigates the dynamic relation between news coverage about older workers and the filing of age discrimination claims by this group while controlling for real-world events (the financial crisis and the debate about the state pension age) and older workers’ expectations of unemployment rates. The findings, which are discussed below, provide new insights regarding the sources of variation in age discrimination claims over time.

Based on the notion of asymmetrical influences of negative news, i.e., Soroka’s (2006) negativity bias, and the premises of Social Identity Theory (Taijfel and Turner 1979), it was anticipated that increased visibility of older workers in the news would increase the number of age discrimination claims. We find support for this assumption: The visibility of older workers in the news media was associated with higher levels of age discrimination claims. This effect occurred with a lag of two quarters, indicating that it takes some time before discriminatory processes emergence as a result of changes in media attention for older workers.

In addition, it was anticipated that increased attention for negative stereotypes in the news media would increase the number of age discrimination claims. The findings offer only support for the influence of a single negative media stereotype: News media’s attention for older workers’ problematic health status was associated with higher levels of age discrimination claims. Previous research shows that concerns about older workers’ health status and associated health insurance premiums hamper managers’ willingness to hire older workers, as they fear an increasing gap between labor costs and productivity (Conen et al. 2011). The here-presented findings suggest that information distributed via the news media may have reinforced negative beliefs about older workers’ health status, with consequences for the extent to which older workers’ report being discriminated.

Surprisingly, we did not find a significant effect of the stereotype that older workers are unproductive. A potential explanation for this null result is that individuals’ personal experiences’ with older workers’ productivity interacted with the influence of the media stereotype. Such individual differences may have canceled out its effect on the aggregate level.

Last, the visibility of the positive media stereotypes that older workers are reliable, highly involved and experienced did not exert an influence on the number of age discrimination claims. The influence of the negative media stereotype that older workers’ health status is problematic remained significant when controlling for positive media stereotypes. This is congruent with previous experimental research, which shows that positive stereotypes about older workers do not offset the effect of negative stereotypes on age discrimination (Krings et al. 2011; Kroon et al. 2016b; Meisner 2012).

Last, and not anticipated, the study shows that older workers’ unemployment expectations negatively influenced the number of age discrimination claims. The experience of discrimination has been shown to elicit fear of being inadequately valued or rejected in the future (Maner et al. 2007; Richman and Leary 2009). One’s fear to encounter future rejections may be heightened when unemployment figures are on the rise—as employment elsewhere is less certain. In times of highly perceived unemployment, older workers may therefore be more inclined to sidestep confrontations with employers—that could potentially lead to unemployment—and refrain from reporting discrimination incidents.

This study has a number of limitations. As the study relies on quarterly data, relatively long time periods are situated between the measurement points. We explicitly aimed to explain macro-level dynamics in age discrimination claims; yet, the relatively high aggregation level comes at the expense of variation at a lower aggregation level. In addition, the study assumed a unidirectional influence of news content on the experience of age discrimination. Yet, it is possible that the reverse relationship holds true. Media coverage about older workers could well be affected by incidents of unfairness or changes in the number of age discrimination claims. We encourage future research to further unravel the underlying dynamics of this relationship.

In sum, the here-presented findings highlight the important role of media in shaping the experience of unequal treatment in the workplace and indicate that a macro-level perspective on the issue can help our understanding of age discrimination dynamics move forward.

## Footnotes

1 Bivariate correlation between the absolute and relative measure of visibility shows that the measures overlap (*r* = 0.94, *p* < 0.001).

2 For example, in the script ‘vita*l.{0,3}’ captured both the word ‘vitaal’ (‘vital;) and ‘vitaliteit’ (‘vitality’).

3 For example, the word ‘oud’ (‘old’) should occur within 10 words of the word ‘werknemer(s)’ (‘employer(s)’) or ‘werker(s)’ (‘worker(s)’) to result in a match. In this way, references to ‘oudere werknemers’ (‘older workers’) were captured, but also more complex formulations resulted in a match, such as the formulation ‘werknemers die ouder worden’ (‘workers who are becoming older’).

4 For example, the stereotype ‘experienced’ was only identified as present in case references to ‘tegenvallende’ (‘disappointing’) or ‘verminderde’ (‘decreasing’) did *not* occur in the same sentence as references to the key terms ‘kennis’ (‘knowledge’) and/or ‘experience’ (‘experience’).

5 One of the independent variables, expected unemployment, is not stationary and should therefore be differenced. However, with differencing a lot of information gets lost. Moreover, if one start working with a model with differenced independent variables, also the dependent variable must be differenced. As results do not differ substantially when differencing the series, the model with non-differenced series is presented.
